# Why do we have a vaccine for measles, but not cytomegalovirus (CMV)?

**DOI:** 10.1371/journal.ppat.1014136

**Published:** 2026-05-04

**Authors:** Mark R. Schleiss, Sallie R. Permar, Stanley A. Plotkin

**Affiliations:** 1 Division of Pediatric Infectious Diseases and Immunology, University of Minnesota, Minneapolis, Minnesota, United States of America; 2 Department of Pediatrics, Weill Cornell Medical Center, New York, New York, United States of America; 3 Department of Pediatrics, University of Pennsylvania, Philadelphia, Pennsylvania, United States of America; 4 Vaxconsult, Doylestown, Pennsylvania, United States of America; University of Iowa, UNITED STATES OF AMERICA

## 1. CMV and measles: Ubiquitous pediatric infections

Cytomegalovirus (CMV) and measles virus (MV) have historically been considered classical, ubiquitous viral infections, usually acquired in childhood. In each case, humans are the only host, although from an evolutionary perspective, MV is a relative “newcomer”, having jumped species from cattle-to-humans (initially as the related virus, *Rinderpest morbillivirus*) in the sixth century [1]. In contrast, all CMVs have co-evolved with their respective animal hosts for millions of years, with human CMV showing the greatest similarity to related primate CMVs [2]. Primary CMV infection is associated with variable clinical manifestations [3,4], but it is often asymptomatic, in striking contrast to the highly symptomatic nature of MV infection. Although CMV infection is commonly subclinical (discussed below), infection acquired during pregnancy can lead to congenital transmission, associated with life-long disability in ~15% of infected newborns [5]. CMV infection also exerts considerable morbidity and mortality in solid organ transplant (SOT) and hematopoietic stem cell transplant (HSCT) patients, and in HIV-positive individuals. In this minireview, we provide an analysis of why, for these two ubiquitous pediatric infections, the development of a measles vaccine was more straightforward than that of a CMV vaccine. Despite virologic and immunobiologic similarities, several key differences exist that impact vaccine-mediated protection. MV infection and disease are prevented by neutralizing antibodies and known cellular mechanisms, whereas protective functions against CMV are more complex and less effective. We have chosen to compare MV and CMV because comparisons between these viruses are particularly timely in view of two key reasons: first, the recent lack of demonstrable vaccine efficacy of a CMV vaccine tested in a Phase 3 study [6], despite major efforts; and secondly, the disturbing re-emergence of MV infection in the United States (US) and globally [7], despite the availability of a safe and effective licensed vaccine.

## 2. The biology, epidemiology, and pathogenesis of CMV differ from measles

Despite some similarities, the biology and epidemiology of CMV are substantially different from MV as summarized in **Table 1**. Both are enveloped viruses that contain virally encoded glycoproteins that elicit antibody responses. However, the viral genomes differ. CMV is a double-stranded DNA (dsDNA) virus, a member of the *Herpesviridae* family [8], while MV is a negative-sense, single-stranded RNA (ssRNA) virus in the *Morbillivirus* genus in the *Paramyxoviridae* family [9]. The modes of acquisition differ, with CMV most commonly requiring intimate exchange of body fluids (blood, breast milk, urine, genitourinary track secretions), while MV is spread by aerosol route. MV is among the most contagious viruses known, while CMV has a low force of infection [10]. Clinical dissimilarities in the manifestation of infections also abound. MV causes a symptomatic disease, rubeola, characterized by cough, conjunctivitis, pneumonitis, and generalized exanthem. Complications include encephalitis, subacute sclerosing pan-encephalitis (SSPE), and death [11]. In contrast, although primary CMV infection occasionally causes disease in the immunocompetent host [12], usually such primary infections are asymptomatic. Thus, vaccination against MV is meant to prevent serious, even life-threatening disease, making the perceived value of a vaccine more intuitively obvious. In contrast, for CMV, immunization is largely driven by the desire to protect the reproductive health of a population – specifically, to protect women of child-bearing age against the risk of transmission of CMV to the developing fetus. Indeed, it has been noted that if a primary CMV infection caused more of a symptomatic ailment, vaccine development may be much more advanced today [13]. In this context, a CMV vaccine is very much analogous to rubella virus (RV) immunization. RV infection was an important teratogen globally, responsible for congenital rubella syndrome (CRS) when acquired by the developing fetus in utero [14]. CRS was solved by development of a successful vaccine in the 1970s, and rubella has since achieved “eradication” status in the Western Hemisphere [15].

**Table 1:** Comparison of biology of cytomegalovirus and measles virus.

Key differences		
	Measles	CMV
Phylogeny	*Paramyxoviridae*	*Herpesviridae*
Genome	ssRNA, negative sense	dsDNA
Replication	Cytoplasmic	Nuclear
Seasonality	Yes; late winter/early spring	No
Communicability	Respiratory droplet; high force of infection; highly communicable; explosive outbreaks	Intimate contact; low force of infection; intimate person-to-person contact
Clinical Disease in Immunocompetent Host	Yes; respiratory infection; conjunctivitis; pneumonia; exanthem; encephalitis	Rare; primary infection usually asymptomatic; disease in congenitally infected fetus, SOT and HSCT, HIV infected individuals
Licensed Vaccine	Yes	No
Strain variants	No; multiple genetic variants, but single serotype and no risk of symptomatic reinfection	Yes; multiple genetic variants with risk of reinfection
Life-long Immunity Post-infection	Yes	No
**Key similarities**		
Virion	Enveloped	Enveloped
Surface proteins	Fusogenic; H and F proteins	Fusogenic; gB
Viremia	Yes	Yes
Immune Response	Neutralizing antibody; cell-mediated immunity	Neutralizing antibody; non-neutralizing antibody (Fc-mediated immune responses [ADCC, ADCP]); cell-mediated immunity
Immune Evasion	Yes; MV infections induce several immunophenotypes • MV infects/depletes T cells and memory B cells • “Immune amnesia” to previous infections and vaccination • Cytokine modulation; predisposition to other infections (Tb)	Yes; CMV infection confers modification of host immune responses • Viral genes promote evasion of immune response to CMV • CMV infection modifies host cytokine, T-cell response; modifies both responses to other pathogens and vaccine responsiveness
Latency	Yes; CNS latency; reactivation leading to SSPE	Yes; frequent subclinical reactivation in immune competent individuals; symptomatic disease upon reactivation in SOT, HSCT, HIV

Despite dissimilarities, CMV and MV share similar structural and immunological motifs (**Table 1**). The envelope glycoproteins of both viruses serve as targets of humoral immune response and are used to enter host cells by a fusion-mediated mechanism. These include, in the case of CMV, glycoprotein B (gB) [16] and other protein complexes, such as gH/gL/gO [17], and for MV, the hemagglutinin [H] and fusion [F] proteins [18]. These glycoproteins provide the basis for the protective immune responses induced by MV vaccine, and (for gB) also serve as targets for candidate CMV vaccines, as considered in more detail below (Section 3).

Regarding the morphology and infectivity of the respective virions, the replication mechanisms of these viruses are very different, with MV replicating its ssRNA genome exclusively in the cytoplasm [19], while CMV replicates its dsDNA genome in the nucleus [20]. Efforts to develop a measles vaccine date back to the 18^th^ century [21], with a series of immunogenic, safe, and highly effective vaccines for MV developed and licensed in the 1970s [22]. A detailed elucidation of MV pathogenesis was not required to develop a vaccine; this was accomplished with a more empirical approach. Post-licensure of the MV vaccine, as the era of modern molecular virology ensued, it became clear that the key correlate of protection was neutralizing antibody generated against viral envelope glycoproteins [23,24]. Development of a CMV vaccine based on the goal of similarly targeting viral envelope glycoproteins might therefore seem to be an intuitive approach to try, but this strategy has so far been only partially successful [25]. Other distinctions between CMV and MV relate to mechanisms of latency and immune evasion. Both viruses are capable of establishing latent infection: for MV, this clinically manifests as SSPE, which may occur decades after a primary infection [26], while for CMV, latency and reactivation occur on a continuous basis, usually with viral shedding occurring (in the absence of symptoms) off-and-on over the life course [27].

Both MV and CMV infections can avoid immune clearance following primary infection. For CMV, many CMV open reading frames (ORFs) encode immunomodulatory genes that negatively interfere with the host response to CMV infection [28]. CMV also modifies the host immune response to other infections and modifies vaccine responsiveness via modulation of cytokine and T cell homeostasis [29]. CMV encodes a plethora of gene products that impair class I and class II MHC function, block antigen presentation, interfere with NK cell responses, and impair antibody-mediated immune control, including antibody-dependent cellular cytotoxicity [28]. MV infection has an even more profound and worrisome impact on host immunity, which has been described as “immune amnesia” and is mediated by virus-induced modification of the host antibody repertoire, and conferred by elimination of memory B and T cells [30]. MV infection also contributes to a general state of immune suppression, associated with elevated interleukin-10 (IL-10), that can lead to reactivation of latent tuberculosis (TB) infection, particularly in children [31]. Although measles infection can have a profound impact on protective immunity to previously acquired pathogens and previously administered vaccines, MV elicits protective autologous lifelong immunity in the overwhelming majority of cases, whereas CMV reinfections occur routinely in immune individuals. It should be noted that immune evasion is a feature of other *Herpesviridae* [32], but in spite of the immunoevasive capacity of this family of viruses, an effective vaccine has been developed against varicella-zoster virus (VZV) [33]. There are, however, major differences [34] in the biology of VZV (an alphaherpesvirus), and CMV (a betaherpesvirus), including the biology of latency and the potential impact of strain variation. VZV variants exist, with a total of up to 9 VZV clades described on the basis of DNA sequence variation [35,36], but there is only a single serotype described, and re-infections in immune individuals are virtually always a manifestation of reactivation of latent infection (herpes zoster), and not re-infection due to acquisition of community-acquired heterologous variants.

In contrast to VZV, CMV clinical isolates demonstrate substantial strain-to-strain variation, particularly in the viral envelope glycoproteins that are the leading candidates for subunit vaccine development [37]. This strain variability might well contribute to re-infection of “immune” individuals in spite of an individual having a past history of CMV (with documented seropositive status) [38,39], including re-infections that occur during pregnancy [39] as well as following SOT and HSCT [40,41], although evidence for this is still being collected and it is a matter where our knowledge is incomplete. This is in contrast to MV, where infection typically confers life-long immunity [42,43] which is protective against re-infection. Re-infections with new strains of CMV occurring during pregnancy appear to be less likely to cause disabilities in the congenitally infected newborn that those that occur during primary antenatal maternal infection [44], and recent data suggest that the majority (77%) of congenital CMV infections that occur in the US are in fact due to primary infections, not re-infections [45]. Nonetheless, the issue of strain variation greatly complicates CMV vaccine development. Having noted this, it is of interest that successful vaccines exist for several viral pathogens that exhibit extensive strain variation, most classically influenza virus [46] and SARS-CoV-2 virus [47]. Although the lack of long-term immunity induced by influenza and COVID vaccines is problematic, requiring frequent re-immunization of individuals as new strains regularly emerge, it is a major concern for a CMV vaccine, which should optimally provide protection for pregnant women throughout their reproductive lives [48]. Thus, the issue of strain variation must be put into the context of what the specific vaccine aims to accomplish. In the context of reproductive health, what is clearly required of a CMV vaccine is the kind of long-term protection that is conferred by a MV vaccine.

## 3. Candidate antigens for a CMV vaccine based on protective immune correlates

A major reason that “CMV is not measles”, particularly with respect to vaccine development, is the relative complexity of the two respective viral proteomes. The MV genome encodes only six major structural proteins, including the F and H proteins. Sequence analyses of MV demonstrate 24 genotypes falling into 8 clades (A-H), but cross-neutralization studies indicate a single serotype, which is compatible with the life-long immunity observed after infection. Immunity to CMV is more complex, and the host correlates of protective immunity are incompletely defined. The phenomenon of re-infection is not only associated with viral strain variation, as noted above, but also the fact that CMV encodes multiple proteins that are responsible for evasion of host immunity. CMV encodes more ORFs than any other human virus. Early evaluation of the CMV genome indicated the presence of 252 ORFs in clinical isolates [49], but more recent transcriptomic analyses of transcriptional start sites, termination sites, and splicing events resulted in annotation of 291 previously undescribed potential coding RNA sequences [50].

Among the antibody, CD4+ and CD8+  T cell arms of immune response which represent the key candidate components of immunity against CMV, it remains unclear what are the requirements for protective immunity, and therefore which are of critical importance to elicit with a vaccine. The answer may depend upon the target population for vaccination, as summarized in **Table 2**. With regard to antibody as a presumed key correlate of protection, adjuvanted subunit gB vaccines have demonstrated moderate efficacy against acquisition of CMV infection in clinical trials in CMV seronegative adolescents and in young adults, as has a disabled, infectious single-cycle (DISC) replication-deficient viral vaccine [25]. An mRNA vaccine expressing two CMV glycoproteins, gB and the “pentameric complex” (PC; a complex consisting of glycoproteins gH and gL, and GPMV proteins UL128, UL130 and UL131), elicited significantly elevated neutralizing antibody titers against CMV, with long-term responses lasting up to 18 months post-immunization, as well as strong T-cell and memory B-cell responses in phase 1 and 2 studies [53]. However, the vaccine failed to demonstrate efficacy when evaluated in a phase 3 study [6]. In contrast to MV vaccine, the virus-neutralizing antibody response may not be the most significant component of the protective IgG response to a CMV vaccine; instead, the non-neutralizing (Fc-mediated) responses, including antibody-dependent cellular cytotoxicity (ADCC) and antibody-dependent cellular phagocytosis (ADCP) [54,55] may be more important. Additionally, and in contrast to MV, CMV transmission is largely mediated by cell-to-cell spread of virus [56]; thus intracellular mechanisms of virus neutralization and pathways that limit cell-to-cell spread of CMV are likely to be important for protection. Live, attenuated vaccines and DISC vaccines add the benefit of inclusion of T cell targets, in particular the ppUL83 (pp65) protein and the IE1/IE2 gene products [25], although a DNA vaccine combining gB and pp65 failed to demonstrate protection against transplant-related CMV disease in a phase 3 study [57].

**Table 2:** Selected active and recent CMV vaccine clinical trials with viral targets of immune response indicated [25,51]. Clinicaltrials.gov study number is shown for active and recent trials.

Vaccine Design	Immunogens	Proposed Protective Mechanism(s)	Target Population and Vaccine Efficacy (VE)
Protein Subunit			
Glycoprotein B (gB) vaccine with MF-59 adjuvant	• gB	• Neutralizing antibody • ADCP • Intracellular neutralization • gB antigenic domain-specific responses	VE of 43%–50% for prevention of CMV acquisition in seronegative adults in two Phase 2 studies
NCT05089630 Adjuvanted subunit glycoprotein vaccine (AS01B adjuvant)	• gB • PC	• Neutralizing antibody	Phase 1 study completed
VLP			
VBI-1501A Enveloped virus-like particle (VLP± alum adjuvant)	• gB	• Neutralizing antibody • Low response to specific gB neutralizing epitope (antigenic domain 2)	Phase 1 study; safety, immunogenicity
VBI-1901 (VLP) Enveloped virus-like particle (VLP ± GM-CSF adjuvant)	• gB • ppUL83 (pp65)	• Neutralizing antibody • pp65-specific T cell response	Phase 2b study
SPYVLP01 (Hepatitis B-platform VLP ± alum or Matrix-M adjuvant)	• PC	• Neutralizing antibody	Phase 1 study completed
mRNA Subunit			
NCT05085366 mRNA platform	• gB • PC	• Neutralizing antibody	Phase 3 study completed in CMV-seronegative adults; VE estimate of 6%–23%; study terminated
NCT05575492 mRNA platform	• gB • PC	• Safety • Immunogenicity	Phase 1-2a study in 9- to 15-year-old children; study terminated
NCT05683457 mRNA platform	• gB • PC	• Safety • Immunogenicity	Phase 2 study in HSCT patients is ongoing
Vectored Vaccine			
NCT06075745^¶^ Modified Vaccinia Virus Ankara (MVA) platform	Triplex • ppUL83 (pp65) • IE1-exon4 (UL123) • IE2-exon5 (UL122)	• Safety • Immunogenicity • VE	Efficacy to be determined by pre-transplant vaccination impact on duration of CMV antiviral therapy in first 100 days post-liver transplant in CMV+/CMV- dyads
Live-Virus/Disabled Infectious Single-Cycle (DISC) Vaccine			
NCT03486834 DISC Vaccine V160 + Merck aluminum phosphate adjuvant [MAPA]	Complete CMV genome in DISC virus; any expressed protein potential immunogen • gB • PC • IE1-exon4 (UL123) • IE2-exon5 (UL122) • Other targets	• Safety • Immunogenicity • VE (infection defined as any positive CMV PCR signal in study participant) • Viremia in vaccinees • Neutralizing antibody • gB specific antibody (ELISA) • T-cell ELISPOT responses to: ◦ Total CMV lysate ◦ ppUL83 (pp65) ◦ IE1	VE of 42.4% for prevention of CMV acquisition in seronegative adults in this study^§^

¶ Other recent studies of Triplex vaccine include NCT02506933, NCT02506933, NCT02506933 (HSCT), NCT05099965 (CMV/HIV co-infection), and NCT05801913 (non-Hodgkin’s lymphoma); see [25] for details.

§ In this study, the serological detection of naturally acquired infections in the context of antibodies induced by V160 vaccination was not feasible; therefore, PCR was used, and, surprisingly, a significant number of placebo recipients were PCR-positive but never developed CMV-reactive antibodies. Thus, some PCR-positive V160 recipients may have been false positives. Based on this assumption and correcting for false positives among the V160 recipients, efficacy increased to 67 or 77% depending on the vaccine group [52].

## 4. The pathway to licensure of a MV vaccine compared to a CMV vaccine

The first measles vaccines (both a live, attenuated vaccine, Rubeovax, and a formalin-activated vaccine, Pfizer-Vax Measles–K) were approved in 1963 based on clinical trials that demonstrated protection against virus acquisition and disease [58]. The complexities of protective immunity against CMV will likely require quite a distinct pathway to vaccine licensure compared to that of MV (**Table 2**), and the pathways (and final licensed products) may differ for prevention of congenital CMV compared to prevention of CMV disease in SOT/HSCT patients. As an example, in the SOT setting, particularly when an organ from a CMV seropositive donor is transplanted into a CMV seronegative recipient, the goal is to prevent virus reactivation from the donor organ. In fact, studies of the subunit gB/MF59 vaccine were only partially successful at achieving a protective effect in this setting. The investigators that performed this study proposed that antibodies induced by the CMV vaccine bind the virus in the donated organ rather than neutralizing cell-free virus, thus reducing subsequent viremia and transmission [59]. However, evidence for protection of SOT patients with other vaccine strategies is quite good. Preventing transmission from an inserted organ, requiring induction of new replication in the cells of the transplant recipient, may be feasible and may succeed via mechanisms that will differ from those induced by vaccines aiming at preventing mucosal acquisition of infection in young women of children-bearing age. SOT studies of a live, attenuated CMV vaccine strain (Towne strain) demonstrated a high level of efficacy against transplant-related CMV disease [60]. The live vaccine approach elicits both antibody and cell-mediated immunity to CMV, whereas other types of vaccines (such as subunit vaccines) may not adequately induce T cell stimulation, but safety concerns limit study of live, attenuated vaccines in the transplant population. To ensure safety while engendering T-cell responses, a modified vaccinia virus Ankara vaccine expressing key CMV T cell antigens - the ppUL83 (pp65) and IE1/2 proteins – has been developed and is in a phase 2 study, the “Control of CMV in Patients Undergoing Liver Transplantation” (COLT) trial [25]. Evaluation of a mRNA vaccine targeting the gB/PC glycoproteins, mRNA-1647, is also ongoing in a phase 2 study in CMV-seropositive HSCT recipients [61], although this vaccine achieved only a 6%–23% efficacy in a recent phase 3 study in CMV-seronegative women of child-bearing age (admittedly this was a very different population [6]). Whether T-cell targets are essential in either or both of these populations needs to be resolved.

The consensus opinion of CMV vaccine manufacturers and experts following a meeting sponsored by the Food and Drug Administration in 2012 was that the primary goal of a preconception CMV vaccine should be to prevent maternal infection [62]. The induction of both B and T cell responses is probably needed to suppress viral replication in the placenta and to reduce CMV transfer to the fetus. Maternal CD4+ T-cells [63] and placental decidual resident memory T cells [64] have been shown to be important in this context. As pre-existing immunity in women is at least partially successful in preventing infection of their fetuses, the goal of a vaccine may be to induce B and T cell responses, similar to or more robust than those elicited by natural infection. In addition to vaccination of adolescents and young women before pregnancy, vaccination of young children [65], with a particular emphasis on children attending group day care, might reduce virus excretion and the risk of exposure of their mothers to CMV during future pregnancies. However, it is clear from studies of natural infection that CMV seropositivity only provides about 70% protection to exposed women, perhaps because of the high risk associated with repeated exposure [66]. Repeated CMV immunization may therefore be needed for women contemplating multiple future pregnancies. Nevertheless, considering the high burden caused by CMV in pregnancy, vaccine prophylaxis is badly needed, even if it is incomplete. Moreover, the likelihood that vaccination could prevent or modify CMV disease in transplant recipients is another attractive indication for vaccine development.

## 5. What are the key recommendations for future studies towards licensure of a CMV vaccine?

A better understanding of the correlates of protective immunity is a major unmet need in planning future CMV vaccine design and studies. Animal models can inform and direct human trials [67] and recent studies have provided new information about the correlates of protection against CMV, particularly at the maternal-fetal interface [68]. Studies of CMV vaccines in children attending daycare centers should be pursued, given the major importance of this environment in dissemination of CMV to the family unit, particular to a pregnant woman or woman contemplating pregnancy. CMV screening, both in the pregnant person and in the newborn infant, will be important not only in raising awareness of CMV, but also in facilitating study of biomarkers that correlate with transmission in pregnancy [69,70] and in informing prognosis in the congenitally-infected infant [71]. Human CMV challenge studies [72] have provided valuable information about the balance between vaccine attenuation and protective immunity, and more should be considered. Future designed phase 3 trials that aim to trigger licensure should not be predicated solely on prevention of acquisition of maternal CMV infection. Rather, if a preconception vaccine impacts the magnitude and duration of maternal CMV viremia/DNAemia, that alone may be sufficient for a licensure endpoint, followed by real-world studies to assess its impact on congenital CMV transmission and/or CMV sequelae in the infant – and these endpoints could be readily monitored pre- and post-licensure by utilizing universal newborn CMV screening programs that are now in place in several US states. By comparison, the parameters for licensure of a CMV vaccine for SOT or HSCT are probably gong to be a combination of other factors, including a reduction in DNAemia, a reduction in the use of antiviral therapy, and reduced transplant complications.

Where do we go next with a CMV vaccine? The MV comparisons are informative and may help point us in the right direction. MV vaccines can confer protection based on neutralizing antibody to envelope glycoproteins, including the hemagglutinin (H) and fusion (F) proteins; CMV immunity is far more complicated [44], and vaccine design will required continued elucidation of the impact of strain variation, viral re-infection, and viral immune evasion mechanisms [6,25,73,51]. Based on the successful MV experience, live attenuated CMV vaccines must be considered [52,74], along with subunit vaccines. Both MV and CMV are common infections, but measles infection and disease are prevented by neutralizing antibodies and known cellular mechanisms, whereas protective functions against CMV are more complex and less effective. Although the focus in recent years has been on subunit antigens that elicit antibody responses, T cell responses are likely required for an effective CMV vaccine [63,75,76], given the fact that neutralizing antibody responses (in contrast to MV) are inadequate. The continued use of animal models of congenital CMV infection, as noted above, will help us design future human clinical trials [67,77]. Most important of all, there must be a public health focus both on the serious impact of MV infection, during a window of time of vaccine hesitancy [7], in addition to continued emphasis on the disabling impact of congenital CMV infection [78]. The most effective response to vaccine misinformation and disinformation is the shared life experiences of families affected by these infections [79,80]. CMV is not measles, but both infections demonstrate the importance of advocacy – not only advocacy for measles prevention using the safe, effective vaccine that we have available now, but also advocacy for both continued research and implementation of strategies that increase public awareness of congenital CMV infection [81].
